# Linkage Disequilibrium, Effective Population Size and Genomic Inbreeding Rates in American Mink Using Genotyping-by-Sequencing Data

**DOI:** 10.3389/fgene.2020.00223

**Published:** 2020-03-13

**Authors:** Karim Karimi, A. Hossain Farid, Mehdi Sargolzaei, Sean Myles, Younes Miar

**Affiliations:** ^1^Department of Animal Science and Aquaculture, Dalhousie University, Truro, NS, Canada; ^2^Department of Pathobiology, University of Guelph, Guelph, ON, Canada; ^3^Select Sires Inc., Plain City, OH, United States; ^4^Department of Plant, Food, and Environmental Sciences, Dalhousie University, Truro, NS, Canada

**Keywords:** American mink, genotyping-by-sequencing, single nucleotide polymorphism, linkage disequilibrium, effective population size, inbreeding rate

## Abstract

Knowledge of linkage disequilibrium (LD) patterns is necessary to determine the minimum density of markers required for genomic studies and to infer historical changes as well as inbreeding events in the populations. In this study, we used genotyping-by-sequencing (GBS) approach to detect single nucleotide polymorphisms (SNPs) across American mink genome and further to estimate LD, effective population size (*Ne*), and inbreeding rates based on excess of homozygosity (F_HOM_) and runs of homozygosity (ROH). A GBS assay was constructed based on the sequencing of *Ape*KI-digested libraries from 285 American mink using Illumina HiSeq Sequencer. Data of 13,321 SNPs located on 46 scaffolds was used to perform LD analysis. The average LD (*r*^2^ ± SD) between adjacent SNPs was 0.30 ± 0.35 over all scaffolds with an average distance of 51 kb between markers. The average *r*^2^ < 0.2 was observed at inter-marker distances of >40 kb, suggesting that at least 60,000 informative SNPs would be required for genomic selection in American mink. The *Ne* was estimated to be 116 at five generations ago. In addition, the most rapid decline of population size was observed between 100 and 200 generations ago. Our results showed that short extensions of homozygous genotypes (500 kb to 1 Mb) were abundant across the genome and accounted for 33% of all ROH identified. The average inbreeding coefficient based on ROH longer than 1 Mb was 0.132 ± 0.042. The estimations of F_HOM_ ranged from −0.44 to 0.34 among different samples with an average of 0.15 over all individuals. This study provided useful insights to determine the density of SNP panel providing enough statistical power and accuracy in genomic studies of American mink. Moreover, these results confirmed that GBS approach can be considered as a useful tool for genomic studies in American mink.

## Introduction

Linkage disequilibrium (LD) refers to the non-random association of alleles at two separate loci within a population (Weir, 1979). The existence of LD between causative variants and genetic markers is the critical assumption of the genomic studies exploring the association between phenotypes and genotypes (Goddard et al., 2016). Knowledge of LD extension is crucial to determine the required marker density to achieve adequate accuracy in both genome-wide association studies (GWAS) and genomic selection (Meadows et al., 2008; Rabier et al., 2016). Moreover, LD patterns across the genome could be affected by evolutionary forces including migration, mutation, genetic drift, natural selection, population structure, and recombination rates (Ardlie et al., 2002). Hence, LD maps are useful tools to study the genetic diversity (McKay et al., 2007) and identify the selective sweeps in livestock populations (Gutiérrez-Gil et al., 2015). In addition, LD information has been frequently used to estimate the effective population sizes (*Ne*) in domestic animals e.g., cattle (Rodríguez-Ramilo et al., 2015), sheep (Prieur et al., 2017), and pig (Tapio and Uimari, 2011). The *Ne* is a useful measure to evaluate the inbreeding levels, historical events, and conservation priorities in animal populations (Theodorou and Couvet, 2006).

Availability of genome-wide markers makes it feasible to estimate the inbreeding levels in the absence of pedigree data (Allendorf et al., 2010). Runs of homozygosity (ROH) were defined as contiguous lengths of homozygous genotypes that transmit identical haplotypes from parents to their offspring (Gibson et al., 2006). Detecting runs of consecutive homozygous segments has been used as an accurate measure to evaluate the inbreeding rates in humans (Kirin et al., 2010), cattle (Ferenčaković et al., 2013; Mastrangelo et al., 2016), and sheep (Purfield et al., 2017). It has also confirmed that ROH could be used as a useful tool to explore the genetic mechanisms of inbreeding depression in the animal populations (Kardos et al., 2016).

American mink (*Neovison vison*) is known as one of the most desired sources of fur in the world. Development of genomic tools can potentially play a prominent role in improving the fur quality, reproductive performance, growth rates, and health traits in American mink breeding programs. Identifying a large number of genetic markers throughout the genome is an essential step to facilitate genomic studies in American mink. Genotyping-by-sequencing (GBS) is a simple and low-cost sequencing-based approach to detect single nucleotide polymorphisms (SNPs) across a reduced subset of genome (De Donato et al., 2013). The GBS-based genotyping has been proved to be highly effective in genomic studies of plants (Deschamps et al., 2012) and animals (Gurgul et al., 2019). Considering the absence of high-throughput SNP panel for American mink, the GBS can be accounted as a cost-effective genotyping method for genomic studies in mink.

Recent advances in next generation sequencing (NGS) technologies have provided a large number of SNPs to study the genetic basis of economically important traits in livestock species. Accordingly, SNPs have been widely used to study LD patterns in domestic animals e.g., cattle (Porto-Neto et al., 2014), sheep (Al-Mamun et al., 2015), pig (Du et al., 2007), and chicken (Pengelly et al., 2016). In addition, these markers are useful tools in assessing the inbreeding levels based on detection of long homozygous regions across the genome (Karimi et al., 2016; Peripolli et al., 2018). The LD method was used to estimate the *Ne* in American mink using microsatellite DNA markers (Lecis et al., 2008; Zalewski et al., 2016), however, no study has been conducted on the extent of LD and detection of ROH in American mink using high-throughput SNP data. Exploring the LD patterns can be an effective step to develop genomic selection and design breeding programs in American mink. Therefore, the main objectives of this study were (1) to estimate the LD levels across the American mink genome using high-throughput SNP makers obtained from GBS data, (2) to evaluate the recent and historical *Ne* based on the pattern of LD decay across the genome, and (3) to estimate the inbreeding levels in the studied population using genomic data.

## Materials and Methods

### Samples, DNA Extraction, and Genotyping-by-Sequencing

Animals were originated from four mink farms in Canada, and were raised at the Aleutian Diseases Research Center (ADRC) (Farid et al., 2015). There was no pedigree data available for all animals to compute the inbreeding coefficients. Genomic DNA was extracted from the spleen tissues of 285 black American mink using the high-salt procedure (Aljanabi and Martinez, 1997). The genomic DNA was digested by the restriction enzyme *Ape*KI (G↓CWGC) which creates a sticky 5′ overhang. At the next step, barcode adaptors unique to each sample along with a standard Y-adaptor were ligated to DNA fragments to generate DNA sequencing libraries. Three separate sets of 96-plex GBS libraries were prepared to amplify DNA fragments by PCR using primers complimentary to the ligated adaptors. Finally, each library was sequenced on a separate lane of Illumina HiSeq Sequencer at Genome Quebec sequencing center to generate single-end reads of PCR products.

### SNP Discovery and Quality Control

The total number of 681,936,405 reads with the length of 100 bp was generated by sequencing platform. As the first step, barcoded reads were demultiplexed into separate files using Sabre software.^1^ Cutadapt software (Martin, 2011) was used to remove primers and adaptor contaminations from sequencing reads, and to discard all reads shorter than 50 bp from the data set. Subsequently, all reads were aligned against the mink genome using maximal exact matches (MEM) algorithm implemented in BWA software (Li and Durbin, 2009). HaplotypeCaller tool from the Genome Analysis Toolkit (GATK) was used to call variants in the aligned reads (McKenna et al., 2010). All variants were filtered based on the following measures suggested by GATK documentation: quality by depth <2.0, mapping quality <40.0, Fisher strand >60.0, mapping quality rank sum test <−12.5, and read position rank sum test <−8.0. Finally, all bi-allelic variants with a minor allele frequency >0.05 and those that occurred in more than 85% of individuals were kept for further analysis. After quality control, a data set including 52,714 SNPs at 260 individuals were remained for further analyses. Only scaffolds which included at least 50 SNPs with the length of >10 Mb were used in the LD analysis.

### Linkage Disequilibrium and Effective Population Size

Two standard LD parameters, *r*^2^ and D′, were computed for all syntenic SNP pairs across the genome using SNP1101 software (Sargolzaei, 2014). The *r*^2^ statistic between two SNPs was calculated using the following equation (Hill and Robertson, 1968):

$$r^{2}=\frac{{\left(P_{A\,b}\,P_{a\,B}-P_{A\,B}\,P_{a\,b}\right)}^{2}}{\left(P_{A}\,P_{B}\,P_{a}\,P_{b}\right)},$$

where P_AB_, P_ab_, P_Ab_, and P_aB_ are the frequencies of the haplotypes AB, ab, Ab, and aB; and P_A_, P_a_, P_B_, and P_b_ are the frequencies of alleles A, a, B, and b in the population, respectively. D′ was computed as explained by Lewontin (1964):

$${\text{D}}^{\prime }=\frac{{\text{D}}_{A\,B}}{{\text{D}}_{m\,a\,x}},$$

where D_AB_ is (P_AB_ – P_A_P_B_) and D_max_ is computed as the min (P_A_P_B_, P_a_P_b_) if D_AB_ < 0 or min (P_A_P_b_, P_a_P_B_) if D_AB_ ≥ 0, respectively.

The distances between syntenic SNP pairs were categorized in three sets including ≤100 kb, ≤1000 kb, and ≤10 Mb, and these sets were classified using bin sizes of 10 kb, 100 kb, and 1 Mb, respectively. The average *r*^2^ was calculated in each bin across all scaffolds and plotted against the median size of bins. We used SNP1101 software to estimate *Ne* based on the observed pattern of LD across the genome. The historical *Ne* was estimated at 200, 100, 50, 10, and 5 generations ago to survey the changes in population sizes.

### Runs of Homozygosity and Inbreeding Rates

Runs of homozygosity were detected using SNP1101 software based on the data of 52,714 SNPs to assess inbreeding levels in the population. The minimum lengths of ROH were defined to be 500 kb, 1 Mb, 2 Mb, 4 Mb, 8 Mb, and 16 Mb in different steps. Minimum window size was set to be 20 SNPs and ROH window was sled one SNP at each time. A genotyping error rate of 0.01 was used to detect ROH segments. The following equation was then used to compute the inbreeding coefficient based on ROH (F_*ROH*_) in each individual (McQuillan et al., 2008):

$$F_{\text{ROH}}=\frac{\sum_{K}\left(L\,e\,n\,g\,t\,h\,\left(R\,O\,H_{K}\right)\right)}{L},$$

where the numerator is the total length of ROHs above a certain length and *L* is the total length of the genome covered by markers, which was 1.75 Gb in the current study. The average of F_*ROH*_ was computed for each length category. In addition, inbreeding coefficient based on excess of homozygosity (F_HOM_) was computed for all individuals using procedure implemented in SNP1101 software.

## Results

### Data Quality Control

The GBS analysis generated 681,936,405 reads with an average of 2,133,590 reads per animal, and a range of 0 to 5,306,590 reads among all samples. On average, 97.12% of reads were mapped to reference genome (Cai et al., 2017). In total, 25 samples did not pass the quality control steps and were discarded from data set. After quality control, 52,714 SNPs on 260 animals remained for further analyses. The average MAF (±SD) was estimated to be 0.185 ± 0.147 and the average of observed heterozygosity (±SD) was 0.284 ± 0.062 among all individuals. The GBS data set of 13,321 SNPs located on 46 scaffolds was used to survey the extension of LD across the American mink genome. These scaffolds covered 720 Mb of whole genome with an average length of 15.7 Mb per scaffold. The number of SNPs per scaffold ranged from 50 (scaffold 24) to 1,320 (scaffold 10).

### Linkage Disequilibrium Patterns and Effective Population Size

Table 1 presents the average *r*^2^ and D′ between adjacent SNPs for all scaffolds. The average distance between two adjacent SNPs was 51 kb across studied scaffolds. The average value of r^2^ ± SD between adjacent SNPs was 0.30 ± 0.35 for all scaffolds and ranged between 0.18 and 0.54 within the scaffolds. On average, 39.09% and 16.97% of all adjacent markers had *r*^2^ > 0.2 and *r*^2^ > 0.8 across the whole genome, respectively. In addition, the average D′ ± SD between adjacent markers was estimated to be 0.79 ± 0.30 with a range of 0.60–0.90 among all scaffolds.

**TABLE 1:** The average *r*^2^ and D′ between adjacent SNPs on each scaffold in American mink genome and the percentage of SNP pairs with *r*^2^ > 0.2 and *r*^2^ > 0.8.

Scaffold number	Scaffold length (Mb)	Number of SNPs	Average *r*^2^ ± SD	Average D′ ± SD	*r*^2^ > 0.2 (%)	*r*^2^ > 0.8 (%)
Scaffold 1	37.08	503	0.33 ± 0.36	0.82 ± 0.29	45.45	19.81
Scaffold 2	29.80	406	0.29 ± 0.37	0.79 ± 0.29	35.65	19.13
Scaffold 3	24.17	456	0.32 ± 0.35	0.83 ± 0.28	44.78	18.26
Scaffold 4	24.19	311	0.25 ± 0.34	0.80 ± 0.28	32.58	14.04
Scaffold 5	23.24	376	0.28 ± 0.34	0.78 ± 0.32	38.50	16.43
Scaffold 6	20.31	758	0.25 ± 0.32	0.77 ± 0.32	34.76	11.76
Scaffold 7	25.40	282	0.34 ± 0.39	0.81 ± 0.32	43.71	22.16
Scaffold 8	26.72	1,234	0.25 ± 0.33	0.80 ± 0.31	33.16	13.29
Scaffold 9	15.31	139	0.42 ± 0.35	0.83 ± 0.30	69.64	21.43
Scaffold 10	22.50	1,320	0.27 ± 0.34	0.79 ± 0.31	36.59	15.41
Scaffold 11	13.42	141	0.25 ± 0.34	0.77 ± 0.34	31.96	14.43
Scaffold 12	13.10	284	0.29 ± 0.34	0.82 ± 0.29	37.85	15.25
Scaffold 13	13.34	50	0.31 ± 0.37	0.74 ± 0.32	42.42	18.18
Scaffold 14	13.56	188	0.31 ± 0.38	0.80 ± 0.31	41.12	21.50
Scaffold 16	16.70	278	0.24 ± 0.32	0.81 ± 0.29	31.51	12.33
Scaffold 17	13.29	56	0.34 ± 0.36	0.89 ± 0.19	48.39	22.58
Scaffold 18	12.91	329	0.26 ± 0.33	0.75 ± 0.33	36.90	12.30
Scaffold 19	12.66	173	0.38 ± 0.39	0.87 ± 0.23	47.44	23.08
Scaffold 20	13.35	104	0.32 ± 0.37	0.81 ± 0.30	42.11	18.42
Scaffold 21	12.91	176	0.26 ± 0.35	0.78 ± 0.30	34.26	12.96
Scaffold 22	13.11	144	0.35 ± 0.37	0.84 ± 0.31	48.00	22.67
Scaffold 23	16.07	79	0.37 ± 0.35	0.81 ± 0.28	54.17	16.67
Scaffold 24	11.92	50	0.31 ± 0.37	0.84 ± 0.21	37.50	18.75
Scaffold 25	23.23	203	0.24 ± 0.35	0.75 ± 0.35	29.92	14.96
Scaffold 26	12.29	226	0.24 ± 0.32	0.82 ± 0.31	30.56	10.42
Scaffold 27	10.49	150	0.25 ± 0.34	0.73 ± 0.34	35.71	14.29
Scaffold 28	12.49	961	0.26 ± 0.33	0.81 ± 0.30	35.29	12.71
Scaffold 29	10.52	126	0.39 ± 0.40	0.74 ± 0.35	46.30	25.93
Scaffold 30	16.12	68	0.23 ± 0.33	0.75 ± 0.32	27.66	12.77
Scaffold 31	12.85	145	0.30 ± 0.35	0.79 ± 0.33	39.56	16.48
Scaffold 32	11.01	116	0.24 ± 0.35	0.76 ± 0.32	28.07	14.04
Scaffold 33	10.69	56	0.24 ± 0.31	0.88 ± 0.24	30.30	12.12
Scaffold 34	10.66	89	0.18 ± 0.28	0.71 ± 0.39	24.07	7.41
Scaffold 36	18.43	1,211	0.25 ± 0.33	0.80 ± 0.30	31.77	13.67
Scaffold 41	16.55	219	0.24 ± 0.34	0.73 ± 0.35	29.92	14.17
Scaffold 45	11.91	85	0.36 ± 0.39	0.88 ± 0.22	45.65	19.57
Scaffold 47	10.04	147	0.36 ± 0.38	0.80 ± 0.33	50.00	21.28
Scaffold 49	10.33	104	0.37 ± 0.41	0.76 ± 0.33	46.67	24.44
Scaffold 64	15.38	203	0.25 ± 0.37	0.73 ± 0.33	30.36	16.96
Scaffold 66	11.16	52	0.19 ± 0.33	0.60 ± 0.42	21.62	10.81
Scaffold 68	10.66	90	0.54 ± 0.41	0.90 ± 0.25	64.52	35.48
Scaffold 70	12.54	215	0.30 ± 0.37	0.83 ± 0.28	36.19	20.00
Scaffold 72	10.47	379	0.28 ± 0.36	0.75 ± 0.34	33.82	17.87
Scaffold 73	13.59	237	0.27 ± 0.34	0.81 ± 0.29	40.13	13.82
Scaffold 93	12.16	189	0.31 ± 0.33	0.79 ± 0.33	46.75	14.29
Scaffold 99	10.59	213	0.32 ± 0.34	0.85 ± 0.24	45.05	16.22
Overall	719.22	13,321	0.30 ± 0.35	0.79 ± 0.30	39.09	16.97

**FIGURE 1:**
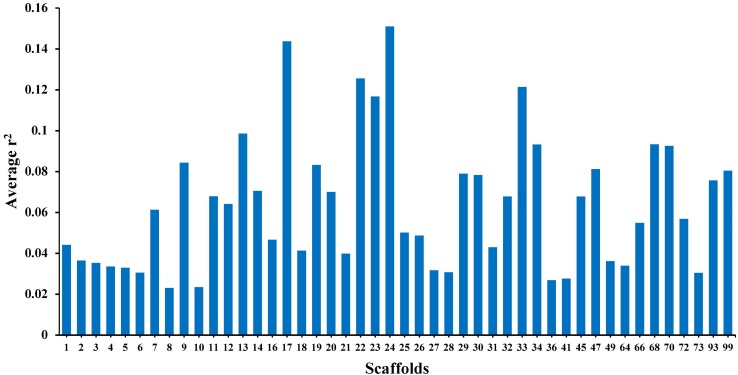
The average *r*^2^ per each scaffold (46 scaffolds) in American mink using GBS data.

**FIGURE 2:**
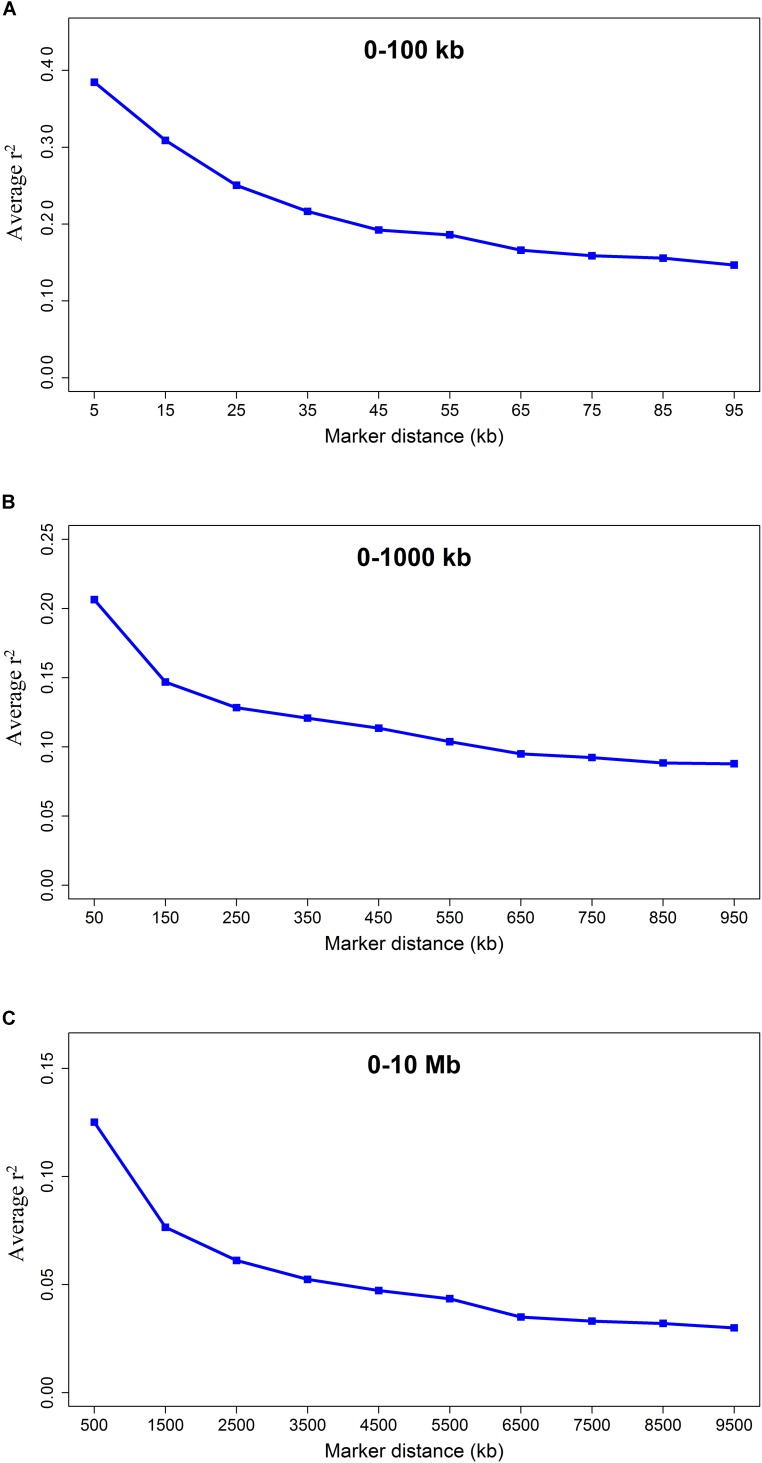
The LD decay represented by the average *r*^2^ for three SNP sets: SNP pairs separated by inter-marker distances of **(A)** 0 up to 100 kb using consecutive 10 kb bins, **(B)** 0 up to 1000 kb using consecutive 100 kb bins, and **(C)** 0 up to 10 Mb using consecutive 1 Mb bins.

The average *r*^2^ fluctuated among various scaffolds (Figure 1). The highest LD level was observed for scaffold 24 (average *r*^2^ = 0.15) whereas the lowest LD level was found on scaffold 8 (average *r*^2^ = 0.02). Table 2 presents the average values of *r*^2^ and D′ as a function of distance between SNP pairs up to 1000 kb. As expected, the average of LD parameters declined with increase in physical distances between markers. Whereas the average *r*^2^ ± SD was 0.38 ± 0.28 in 0–10 kb inter-marker distances, it was reduced to 0.08 ± 0.12 in marker interval of 900–1000 kb. Similar trend was also observed for D′ values over different inter-marker distances (Table 2). Furthermore, the average *r*^2^ was computed by dividing all syntenic SNP pairs into marker intervals spanning up to 100 kb (using bins of 10 kb), 1000 kb (using bins of 100 kb), and 10 Mb (using bins of 1 Mb). The average *r*^2^ of consecutive bins was presented for three distance sets in Figure 2.

**TABLE 2:** Average *r*^2^ and D′ ± SD over physical distances up to 1000 kb, pooled over all scaffolds, in American mink.

SNP pairs distance (kb)	LD parameters		
	Number of pairs	Average *r*^2^ ± SD	Average D′ ± SD
0–10	9,024	0.38 ± 0.28	0.79 ± 0.27
10–20	4,659	0.30 ± 0.23	0.73 ± 0.28
20–30	4,513	0.25 ± 0.22	0.72 ± 0.28
30–40	4,055	0.21 ± 0.21	0.71 ± 0.28
40–50	4,355	0.19 ± 0.20	0.71 ± 0.29
50–60	4,163	0.18 ± 0.20	0.70 ± 0.29
60–70	4,160	0.17 ± 0.19	0.70 ± 0.29
70–80	4,110	0.16 ± 0.19	0.68 ± 0.29
80–90	4,164	0.15 ± 0.18	0.67 ± 0.29
90–100	3,980	0.14 ± 0.18	0.67 ± 0.28
100–200	38,171	0.14 ± 0.17	0.66 ± 0.31
200–300	35,858	0.13 ± 0.17	0.66 ± 0.31
300–400	34,748	0.12 ± 0.16	0.64 ± 0.31
400–500	34,209	0.11 ± 0.16	0.61 ± 0.32
500–600	33,502	0.10 ± 0.14	0.60 ± 0.32
600–700	33,036	0.09 ± 0.14	0.57 ± 0.32
700–800	32,085	0.09 ± 0.13	0.56 ± 0.32
800–900	30,318	0.08 ± 0.13	0.55 ± 0.32
900–1000	29,765	0.08 ± 0.12	0.55 ± 0.32

**FIGURE 3:**
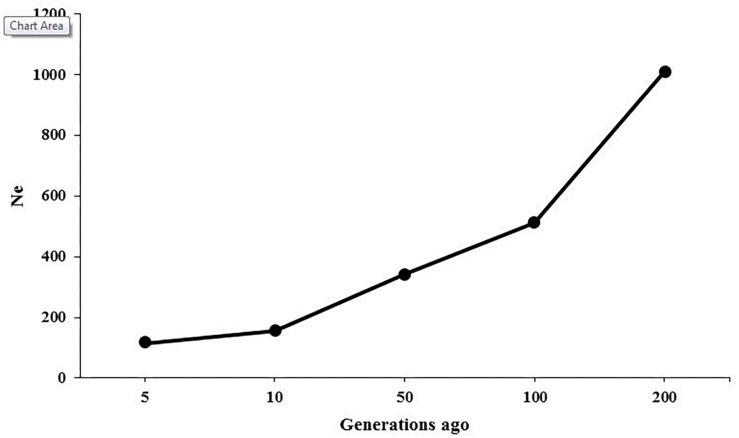
Effective population sizes (Ne) in American mink as a function of generations.

The ancestral and recent *Ne* were inferred based on the relationship between LD patterns across various marker intervals and the size of population at different generations. Figure 3 presents the changes in historical *Ne* from 200 to 5 generations ago, showing that the estimate of *Ne* was 1,012 at 200 generations ago and was reduced to 116 at 5 generations ago. The Ne was predicted to be 104 for present generation using regression model.

### Runs of Homozygosity and Inbreeding Rates

**FIGURE 4:**
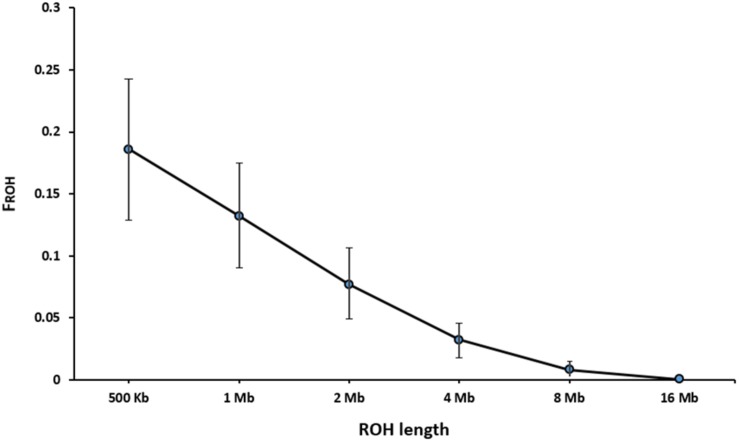
Trend of F_ROH_ based on the cut-off length categories for defining ROH.

Table 3 presents the average length and the number of segments identified in each ROH length category. The average number of ROH (±SD) identified per individual was 216 ± 56 with an average length of 2.15 ± 2.00 Mb for the length threshold of 500 kb. Short segments (500 kb to 1 Mb) composed the main percentage of total number of detected ROH (33%). The longest autozygous segment was found on scaffold 2 with the length of 22.3 Mb (245 SNPs). The highest number of ROH per scaffold (1500 segments) was observed for scaffold 8 and tended to decline with scaffold length. Figure 4 represents the trend of F_ROH_ as a function of criterion lengths from 500 kb to 16 Mb. While the average of F_ROH_ > 500 kb was 0.186 ± 0.056 among all samples, the values of F_ROH_ for segments >8 Mb tended to be zero. F_ROH_ values were in range of 0.02 to 0.36 among individuals. The most inbred animal exhibited 365 ROH >500 kb with an average length of 2.59 Mb per segment. The minimum number of ROH >500 kb detected per individual was 40 with an average length of 1.91 Mb.

**TABLE 3:** Descriptive statistics of runs of homozygosity (ROH) in different length categories: average number of ROH per individual, average length per ROH class, minimum and maximum number of ROH detected per individual, cumulative frequency of each ROH class, and correlation coefficient between F_ROH_ and F_HOM_ in each length category.

ROH class	Average number of ROH per individual ± SD	Average ROH length (Mb) ± SD	Minimum number of ROH per individual	Maximum number of ROH per individual	Cumulative frequency (%)	Correlation coefficient with F_HOM_
ROH > 500 kb	216 ± 56	2.16 ± 2.00	40	332	100	0.87
ROH > 1 Mb	115 ± 44	2.87 ± 2.11	22	241	67	0.84
ROH > 2 Mb	84.50 ± 25	4.09 ± 2.20	8	155	36.5	0.78
ROH > 4 Mb	31 ± 10.90	6.30 ± 2.22	2	71	13.35	0.68
ROH > 8 Mb	5.60 ± 2.90	10.08 ± 2.12	0	16	2.35	0.49
ROH > 16 Mb	0.08 ± 0.29	17.65 ± 2.90	0	2	0.34	0.12

Genomic inbreeding coefficient was estimated based on excess of homozygosity (F_HOM_) for all individuals. The estimates ranged from −0.44 to 0.34 among different samples and had an average of 0.15 over all individuals. High correlation (0.87) was observed between F_ROH_ (minimum length > 500 kb) and F_HOM_ across the studied samples. However, the correlation coefficients were declined along with increase in the ROH lengths (Table 3).

## Discussion

We used GBS analysis to detect SNPs across the genome and investigate the LD extensions in American mink. Despite the lack of chromosome-scale genome assembly and being far from the availability of standard functional and structural annotation of American mink genome, it was still possible to use the reference genome for discovering SNP markers using GBS technique. The high percentage (97.12%) of reads aligned to reference genome indicated that library preparation and optimization steps were successfully performed. In this study, GBS analysis provided 52,714 SNPs across 895 scaffolds for genomic studies in American mink. Similarly, Cai et al. (2018) obtained 34,816 SNPs in 2,451 individuals using GBS data to study the association of SNP markers with body size and pelt length in American mink. The greater number of SNP markers extracted in the current study compared to those identified by Cai et al. (2018) can be mainly due to differences in the restriction enzymes and read depths applied in the two studies. However, in both studies, the highest density of SNPs was located on scaffolds 10 and 8. The GBS analysis also generated a large number of SNPs in other livestock species including horse (30,429), cattle (13,396) and sheep (57,377), which were subsequently applied in revealing the genetic differentiation of the studied populations (Gurgul et al., 2019). Moreover, GBS data have widely been used to investigate the extension of LD in other species e.g., fox (Johnson et al., 2015), olive cultivars (D’Agostino et al., 2018), and cultivated oat (Huang et al., 2014). The results of the present study confirmed that GBS could be a valuable tool for SNP discovery in American mink.

The average LD between adjacent markers was measured using *r*^2^ and D′. The average LD (*r*^2^ ± SD) between adjacent SNPs was 0.30 ± 0.35 over all scaffolds with a high percentage (39.09%) of *r*^2^ > 0.2 between markers (Table 1). Cai et al. (2018) reported a range of 0.2 to 1 for *r*^2^ values between adjacent markers in a genome-wide association study of body size and pelt length in American mink, which are in agreement with our results. Furthermore, the extent of LD was measured by computing the average *r*^2^ for all SNP pairs up to 10 Mb using three distance sets (Figure 2). A rapidly decreasing trend was observed for *r*^2^ values at shorter distances (≤40 kb). However, the LD gradually decayed at longer inter-marker distances and no remarkable change was observed at longer than 6 Mb distances (Figure 2C). The extension of LD across the genome is a critical parameter to determine the number of markers required to achieve a reasonable accuracy in both GWAS and genomic selection. In this regard, the *r*^2^ > 0.2 between a marker and QTL was assumed as the critical threshold to obtain an accuracy of >0.80 in genomic selection studies (Hayes et al., 2003; Meuwissen, 2009). In addition, the *r*^2^ > 0.3 is suggested to achieve enough statistical power in GWAS (Ardlie et al., 2002). In the present study, the average LD (*r*^2^) declined to <0.2 at inter-marker distances of >40 kb, suggesting that at least 60,000 informative SNPs (2.4 Gb/40 kb, where 2.4 Gb is the size of genome assembly) would be required to capture useful LD information necessary for genomic selection. Furthermore, given that the average LD (*r*^2^) decayed to <0.3 at inter-marker distances of >20 kb, the minimum of 120,000 SNP markers would be required for GWAS in American mink (2.4 Gb/20 kb). The average distance of 51 kb between adjacent markers implied that 13,321 SNPs would not provide appropriate accuracy in GWAS and genomic selection for American mink. To our knowledge, this was the first study to determine the patterns of LD decay across the American mink genome. However, Gautier et al. (2007) and Khatkar et al. (2008) suggested that 75,000 to 300,000 SNPs would be required for association mapping studies within worldwide cattle breeds, which is in agreement with the density suggested in the present study for American mink. For instance, similar to our results, *r*^2^ > 0.3 extended up to distances of 10–20 kb in Nguni and Drakensberger cattle breeds of South Africa (Makina et al., 2015) and several cattle breeds of Iran (Karimi et al., 2015). In addition, short extensions of LD (average *r*^2^ > 0.3 at distances of >20 kb) were reported at some breeds of sheep (Kijas et al., 2014; Chitneedi et al., 2017; Liu et al., 2017), pig (Amaral et al., 2008; Ai et al., 2013), and domestic cats (Alhaddad et al., 2013), which are comparable to the ranges obtained in our study.

The LD-based estimations of *Ne* in American mink showed that the population size was approximately 1,012 at 200 generations ago and was decreased to 116 at five generations ago (Figure 3). A relatively steep decline was observed in the population size between 200 and 100 generations ago, which can be attributed to typical reduction of population size due to domestication process. It seems that the earliest breeding of American mink in captivity was initiated approximately 150 years ago in 1866 for producing fur (Bowness, 1996), which falls into the estimated time of the decrease in population size. Zalewski et al. (2016) estimated the population sizes of 17.5 to 78.8 using microsatellite DNA in American mink on the Swedish coasts. Furthermore, the *Ne* was estimated to be 7.2–34.8 among mink populations in Spain using microsatellite DNA markers (Lecis et al., 2008). These discrepancies may result from differences in the type and number of molecular markers, genetic backgrounds of the studied populations, and the conservation programs applied at European countries to control the population size of American mink as an invasive species.

We defined ROH as the homozygous segments with different sizes (>500 kb to > 16 Mb). The average level of autozygosity was obtained to be 0.132 ± 0.042 on the basis of ROH longer than 1 Mb (Figure 4). Although there is no evidence on inbreeding rates based on ROH in American mink, a wide range of F_ROH_ estimations were reported in other domestic animals, depending on autozygoisty level and historical background of the populations. For instance, Ferenčaković et al. (2013) observed the range of 0.087–0.156 for F_ROH_ > 1 Mb in four cattle breeds. A similar range (0.026–0.190) was reported in six Chinese goat breeds (Islam et al., 2019). A slightly higher F_ROH_ > 1 Mb (0.168 ± 0.052) was estimated for Jinhua Pigs using sequencing data (Xu et al., 2019). On the other hand, lower estimation of 0.084 ± 0.061 was observed in the study on Valle del Belice sheep on the basis of ROH > 1 Mb (Mastrangelo et al., 2017). The differences in estimations of inbreeding rates derived from ROH could also be raised by uneven marker densities applied in various studies (Ceballos et al., 2018).

Our results showed that short extensions of homozygous genotypes (500 kb to 1 Mb) were abundant across the genome and accounted for 33% of all ROH identified. However, only a small number of the long ROH segments (≥ 8 Mb) was detected in this study (Table 3). Whereas long ROH are most likely observed in the genomic regions with low recombination rates and selective sweeps, small segments tend to be found at short haplotype blocks which are highly associated with high LD regions (Gibson et al., 2006). Detection of larger number of short ROH in this study could be attributed to the fact that while high level of LD (average *r*^2^ = 0.30) was observed between adjacent markers, the extension of medium LD (*r*^2^ > 0.2) was restricted to short inter-marker distances (≤40 kb). In addition, it is likely that the total number and length of ROH obtained for long segments (≥ 8 Mb) in the current study were underestimated due to short extension of scaffolds, which restricted the detection of longer ROH.

The extension of ROH can be also useful to infer the recent and ancient inbreeding events in the populations. Longer ROH are associated with recent inbreeding in the population because recombination rates would not be enough to break up long segments over a few generations. On the other hand, short tracts of homozygosity tend to be correlated with ancient inbreeding (Pemberton et al., 2012). The abundance of short ROH (< 1 Mb) in this study was in accordance with the intense reduction of population size between generation 100 and 200 (Figure 3). However, lack of chromosome-scale information and the existence of small scaffolds restricted the detection of longer ROH in this study and made it impossible to infer recent inbreeding rates.

The inbreeding rate of the studied population based on excess of homozygosity was estimated to be in a range of −0.44 to 0.34 among all individuals with an average of 0.15 over all samples. This moderate degree of inbreeding is to some extent due to positive assortative mating, which is commonly used in the mink farms. Demontis et al. (2011) reported a range of 0.02–0.29 of inbreeding rates in black American mink using DNA microsatellite markers, which was in agreement with the range obtained in the current study. However, Belliveau et al. (1999) reported a higher degree of inbreeding rate (0.271) within samples in farm and wild American mink using DNA microsatellite markers, which was attributed to linebreeding and using related males in the studied populations. In contrast, Thirstrup et al. (2015) estimated significant negative F_*IS*_ (−0.150 to 0.005) using a panel of 194 SNPs, which indicated that farms were successful in preventing inbreeding. These discrepancies might be also due to the differences in number and nature of markers, and approaches used in various studies. Furthermore, F_HOM_ values were highly correlated (0.87) with F_ROH_ (minimum length > 500 kb) across the studied samples. In accordance with the value estimated in this study, high correlations of 0.83–0.95, 0.89–0.99, 0.91–0.98 and 0.89 were also reported in Italian local cattle breeds (Mastrangelo et al., 2016), Chinese goat breeds (Islam et al., 2019), six commercial sheep breeds (Purfield et al., 2017) and four dairy cattle breeds (Ferenčaković et al., 2013), respectively. High correlation observed between F_ROH_ and F_HOM_ suggested that the proportion of ROH regions could be an accurate estimator to reveal the inbreeding rates in the population.

Genomic selection can be developed as an efficient breeding strategy to improve the economically important traits in the mink industry (Karimi et al., 2019). Accessibility to more accurate genome assembly, designing commercial SNP panel, and collecting reliable phenotypes would be essential steps to achieve this goal in the mink industry. The results of this study can be helpful to determine the minimum distance between markers required for designing the SNP panel. Furthermore, genomic data would be useful to control the inbreeding rates and determine the genetic structure of mink populations.

## Conclusion

This study provided the first draft of LD patterns across the American mink genome using GBS data. The estimated *r*^2^ > 0.2 extended up to inter-marker distances of 40 kb suggesting that at least 60,000 SNPs would be required to achieve adequate accuracy in genomic selection programs of American mink. A decreasing trend of effective population size was observed from 200 to 5 generations ago. The LD levels and ROH patterns across the genome indicated that the most rapid decline in population size occurred between 100 and 200 generations ago. Short extensions of homozygous genotypes (500 kb to 1 Mb) were abundant across the genome and were used to infer the levels of ancient inbreeding. However, the chromosomes-scale information is necessary to infer the recent inbreeding events in American mink populations. This study provided useful insights to determine the density of SNP panel providing enough statistical power and accuracy in genomic studies of American mink. Moreover, these results confirmed that GBS approach can be considered as a useful tool for genomic studies in American mink.

## Data Availability Statement

The datasets generated for this study can be found in the FigShare Repository: doi: 10.6084/m9.figshare.9757784.

## Ethics Statement

The animal study was reviewed and approved by the Institutional Animal Care and Use Committee.

## Author Contributions

KK and YM carried out the statistical analysis and interpreted the data. AF and SM collected the genetic materials and carried out the laboratory analyses. AF and YM conceived the study and participated in its design and coordination. KK and YM wrote the manuscript. MS provided the software and supported the statistical analysis. YM and AF reviewed the manuscript. All authors read and approved the final manuscript.

## Conflict of Interest

MS was employed by Select Sires Inc. The remaining authors declare that the research was conducted in the absence of any commercial or financial relationships that could be construed as a potential conflict of interest.

## Footnotes

1 https://github.com/najoshi/sabre
